# Beyond Bone Mineral Density: Real-World Fracture Risk Profiles and Therapeutic Gaps in Postmenopausal Osteoporosis

**DOI:** 10.3390/diagnostics15151972

**Published:** 2025-08-06

**Authors:** Anamaria Ardelean, Delia Mirela Tit, Roxana Furau, Oana Todut, Gabriela S. Bungau, Roxana Maria Sânziana Pavel, Bogdan Uivaraseanu, Diana Alina Bei, Cristian Furau

**Affiliations:** 1 Multidisciplinary Doctoral School, “Vasile Goldis” Western University of Arad, 310414 Arad, Romania; anamaria.ardelean27@gmail.com (A.A.); furau.cristian@uvvg.ro (C.F.); 2 Doctoral School of Biological and Biomedical Sciences, University of Oradea, 410087 Oradea, Romania; gbungau@uoradea.ro (G.S.B.); pavel.mariaroxanasanziana@student.uoradea.ro (R.M.S.P.); 3 Department of Pharmacy, Faculty of Medicine and Pharmacy, University of Oradea, 410028 Oradea, Romania; 4 Department Medicine, Faculty of Medicine, “Vasile Goldis” Western University of Arad, 310414 Arad, Romania; 5 Department of Pathophysiology, Faculty of Medicine, “Vasile Goldis” Western University of Arad, 310414 Arad, Romania; bisorcaoanacristina@yahoo.com; 6 Department of Psycho-neurosciences and Recovery, Faculty of Medicine and Pharmacy, University of Oradea, 410073 Oradea, Romania; buivaraseanu@uoradea.ro; 7 Department of Medical Disciplines, Faculty of Medicine and Pharmacy, University of Oradea, 410073 Oradea, Romania; diana.bei@didactic.uoradea.ro

**Keywords:** postmenopausal osteoporosis, bone mineral density, DXA, fracture risk, treatment gap

## Abstract

**Background/Objectives**: Osteoporosis remains a leading cause of morbidity in postmenopausal women, yet many high-risk individuals remain undiagnosed or untreated. This study aimed to assess the prevalence of osteoporosis and osteopenia, treatment patterns, and skeletal fragility indicators in a large cohort of postmenopausal women undergoing DXA screening. **Methods**: We analyzed data from 1669 postmenopausal women aged 40–89 years who underwent DXA evaluation. BMD status was categorized as normal, osteopenia, or osteoporosis. Treatment status was classified based on active antiosteoporotic therapy, calcium/vitamin D supplementation, hormonal therapy (historical use), or no treatment. Logistic regression models were used to explore independent predictors of osteoporosis and treatment uptake. **Results**: A total of 45.0% of women had osteoporosis and 43.5% had osteopenia. Despite this, 58.5% of the population, over half of women with osteoporosis, were not receiving any active pharmacologic treatment. Bisphosphonates were the most prescribed therapy (17.9%), followed by calcium/vitamin D supplements (20.6%). A prior history of fragility fractures and radiological bone lesions were significantly associated with lower BMD (*p* < 0.05). Historical hormone replacement therapy (HRT) use was not associated with current BMD (*p* = 0.699), but women with HRT use reported significantly fewer fractures (*p* < 0.001). In multivariate analysis, later menopause age and low BMD status predicted higher odds of receiving active treatment. **Conclusions**: Our findings highlight a substantial care gap in osteoporosis management, with treatment primarily initiated reactively in more severe cases. Improved screening and earlier intervention strategies are urgently needed to prevent fractures and reduce the long-term burden of osteoporosis.

## 1. Introduction

Osteoporosis is a systemic skeletal disease marked by reduced bone mass and compromised microarchitecture, leading to increased bone fragility and susceptibility to fractures. It affects over 200 million individuals globally, the majority of whom are postmenopausal women [1]. Following menopause, the abrupt decline in estrogen levels triggers a rapid phase of bone loss, particularly within the first decade [2,3]. Although the clinical definition of osteoporosis is based on a dual-energy X-ray absorptiometry (DXA) T-score of ≤−2.5 at the lumbar spine or hip [4], numerous studies have shown that a substantial proportion of fragility fractures occur in women with osteopenic T-scores (between −1.0 and −2.5). In fact, up to one in three women over the age of 50 will experience an osteoporotic fracture during their lifetime [5].

From a pathophysiological perspective, estrogen plays a crucial role in maintaining bone homeostasis [6]. It inhibits osteoclast-mediated bone resorption and promotes osteoblast survival, thereby preserving trabecular integrity. The estrogen withdrawal after menopause accelerates osteoclast genesis and bone turnover, culminating in a net loss of bone mass and structural quality [7]. As a result, postmenopausal women enter a period of heightened skeletal vulnerability.

Current guidelines recommend DXA as the gold standard for osteoporosis diagnosis and risk stratification [4,8]. Tools such as FRAX integrate clinical risk factors and BMD to predict 10-year fracture risk [9]. Treatment strategies focus on reducing fracture incidence through antiresorptive therapies (e.g., bisphosphonates, denosumab) or anabolic agents (e.g., teriparatide, romosozumab), both of which have demonstrated robust effects on BMD and fracture prevention [10]. Menopausal hormone therapy (MHT) may also confer skeletal benefits, especially in the early postmenopausal years, although its broader use is limited by cardiovascular and oncologic safety considerations [3].

Despite these advancements, significant gaps remain in the detection and management of osteoporosis. Many women at high risk, particularly those with prior fractures or low BMD, remain undiagnosed or undertreated. Moreover, treatment tends to be reactive rather than preventive, often initiated only after a fracture has occurred. Understanding the real-world patterns of skeletal fragility indicators and treatment uptake is critical for improving secondary prevention and optimizing long-term outcomes [11,12].

The present research evaluated the skeletal risk indicators and treatment patterns in a large cohort of postmenopausal women undergoing DXA screening, with a particular focus on fragility fractures, radiological bone lesions, and prior hormone replacement therapy. By analyzing both densitometric and clinical data, we sought to identify gaps in care and predictors of undertreatment in this vulnerable population.

## 2. Materials and Methods

### 2.1. Study Design and Participants

This observational cross-sectional study included all postmenopausal women evaluated for bone mineral density (BMD) by dual-energy X-ray absorptiometry (DXA) in the County Emergency Clinical Hospital Arad between 2017 and 2021. A total of 1669 women aged 40 to 89 years (mean age: 63.1 ± 8.7 years; median: 63) were included. Menopause was defined as the absence of menstruation for at least 12 consecutive months. Both natural and surgically induced menopause (bilateral oophorectomy) were accepted, acknowledging that women with surgical menopause may enter the postmenopausal state at a younger age. Consequently, the inclusion of women aged 40–49 reflects real-world clinical scenarios where early or abrupt estrogen loss necessitates earlier DXA screening. Patients with secondary osteoporosis due to severe endocrine or metabolic conditions (e.g., active primary hyperparathyroidism or osteomalacia) were excluded, while common comorbidities such as thyroid dysfunction or type 2 diabetes were retained for analysis.

### 2.2. Clinical and Demographic Data

Demographic and clinical data were extracted from electronic medical records and included age, body mass index (BMI), and area of residence (urban or rural). BMI was calculated using recorded weight and height at the time of DXA evaluation. Menopausal parameters considered relevant to bone status included age at menopause and type of menopause (natural vs. surgical).

The presence of selected comorbidities was recorded due to their known influence on bone metabolism: thyroid disorders (hypo- or hyperthyroidism, based on physician-confirmed diagnosis and clinical documentation), type 2 diabetes or prediabetes, and oncological history (e.g., breast or ovarian cancer). We also noted chronic corticosteroid exposure (e.g., for asthma or rheumatologic disease) and prior chemotherapy. Past use of hormone replacement therapy (HRT) was recorded as a binary variable, acknowledging its possible residual impact on bone density despite current discontinuation.

Bone mineral density (BMD) was evaluated at the lumbar spine (L1–L4) and femoral neck using standard dual-energy X-ray absorptiometry (DXA) performed in a certified outpatient clinic, with calibrated equipment and licensed technicians. All scans followed standardized acquisition and positioning protocols recommended by the International Society for Clinical Densitometry (ISCD) [13]. According to World Health Organization (WHO) criteria [14], T-scores were interpreted as follows: osteoporosis (T ≤ −2.5), osteopenia (T between −1.0 and −2.4), and normal BMD (T ≥ −1.0). For each patient, the lowest T-score across the measured sites was used to define final BMD status. This “lowest value” rule reflects clinical practice and prioritizes fracture risk stratification. For subsequent analysis, BMD diagnosis was recorded both as a three-level categorical variable (normal, osteopenia, osteoporosis) and as a binary variable (osteoporosis: yes/no).

### 2.3. Classification of Skeletal Risk Indicators (Fractures, Bone Lession, and Family History)

Personal fracture history was classified based on self-reported data and available medical records, focusing on fractures that occurred after the age of 50 and resulted from low-energy trauma (e.g., falls from standing height). Anatomical sites were consolidated into ten categories: hand/fingers, foot/ankle, hip/pelvis, leg/knee, ribs, shoulder/clavicle, upper arm, wrist/forearm, vertebral fractures, and other/multiple for non-specific or combined reports. Participants without any reported fractures were coded as no fracture.

Family history of fragility fractures was defined as the presence of hip, vertebral, or wrist fractures in first-degree relatives. These were recorded according to the reported anatomical site.

Bone lesions were identified based on available radiology notes or clinical documentation, including indications of vertebral compressions, degenerative changes, or other skeletal abnormalities. These imaging studies were not performed systematically as part of the study protocol but were conducted during routine clinical care prior to or around the time of the DXA evaluation, in response to specific symptoms or clinical suspicion. The most common indications for imaging included chronic back pain, localized joint pain, recent low-energy trauma, or suspected vertebral compression. Findings included vertebral deformities (e.g., wedge/compression fractures), cortical thinning, degenerative changes, and other skeletal abnormalities. All available imaging data were reviewed and categorized according to anatomical location: vertebral, upper extremity, lower extremity, multiple sites, or unspecified. This classification aimed to preserve clinical relevance and comparability across patients with diverse radiographic findings. The full breakdown of lesion categories is provided in Table 1.

### 2.4. Antiosteoporosis Treatment Categories

Treatment exposure at the time of DXA evaluation was categorized into six groups based on the most potent agent reported. A complete list of representative agents for each treatment category is shown in Table 2. For participants who had used more than one type of intervention, categorization was based on therapeutic potency, with priority given to antiresorptive agents (e.g., bisphosphonates over supplements or HRT).

### 2.5. Statistical Analysis

All data were initially compiled and cleaned in Microsoft Excel, then imported into JASP (version 0.19.3) for statistical analysis. Descriptive statistics, including means, standard deviations, frequencies, and percentages, were used to summarize the demographic and clinical characteristics of the study population. Group comparisons (e.g., across treatment categories, BMD status, or fracture presence) were assessed using chi-square (χ^2^) tests. A *p*-value < 0.05 was considered statistically significant.

To identify independent predictors of osteoporosis and active treatment uptake, multivariate logistic regression models were constructed. Covariates included age at menopause, body mass index (BMI), area of residence, personal and family history of fractures, presence of radiological bone lesions, hormone replacement therapy (HRT), prior corticosteroid or chemotherapy exposure, and DXA diagnosis. Results were reported as odds ratios (ORs) with 95% confidence intervals (CIs), and model performance was evaluated using AIC, deviance, and Nagelkerke R^2^.

Associations between HRT and skeletal fragility indicators, including fragility fractures, bone lesions, and BMD categories, were explored using contingency tables and chi-square tests, supported by visual figures for key predictors where appropriate.

### 2.6. Ethical Onsiderations

The study was approved by the Ethics Committee for Clinical Studies of the County Emergency Clinical Hospital Arad, approval No. 87, dated 13 June 2025. All procedures were conducted in accordance with institutional regulations, the principles of the Declaration of Helsinki, and current ethical standards for medical research involving human participants. All data were anonymized prior to analysis and used solely for research purposes, in compliance with applicable confidentiality and data protection laws. No additional interventions beyond standard diagnostic and treatment protocols were performed, and informed consent was obtained where applicable.

## 3. Results

### 3.1. Baseline Characteristics

**Figure 1:**
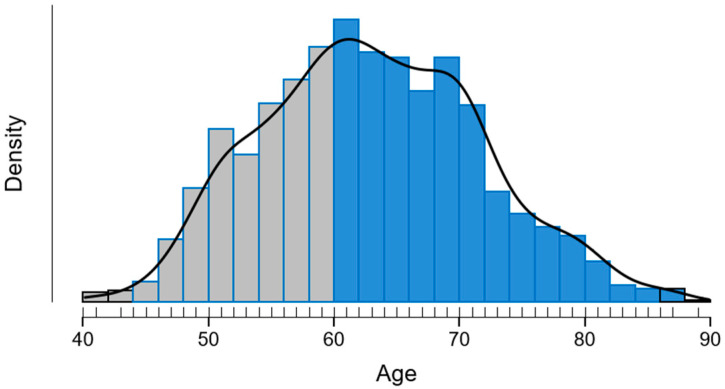
Histogram and density curve of participant age distribution. Gray bars indicate ages ≤ 60 years; blue bars indicate ages > 60 years.

A total of 1669 postmenopausal women, aged between 40 and 89 years (mean age 63.1 ± 8.7 years; median 63), were included in the analysis. The age distribution was slightly right-skewed but visually approximated a normal curve, as shown in Figure 1. Although the Shapiro–Wilk test indicated a statistically significant deviation from normality (*p* < 0.001), this result likely reflects the large sample size, and the histogram supports a near-normal distribution.

The largest subgroup was aged 60–69 years (40.4%), followed by 50–59 (30.8%) and 70–79 (20.0%). Only 5% of participants were younger than 50, and 4% were 80 years or older (Table 3).

Most participants (69%) were from urban areas, while 31% resided in rural settings. The mean body mass index (BMI) was 28.8 ± 5.5 kg/m^2^, reflecting a predominantly overweight population. Specifically, 76% of the women had a BMI ≥ 25 kg/m^2^, indicating overweight or obesity, while only 24% had a BMI within the normal range (18.5–24.9 kg/m^2^). Regarding medical comorbidities, 8% of participants reported thyroid disorders, and 15% had either type 2 diabetes mellitus or prediabetes. A history of cancer (including breast or ovarian malignancies) was present in 4% of the cohort. Additionally, 5% were receiving or had received chronic corticosteroid therapy, primarily for rheumatic diseases or bronchial asthma, while 3% had undergone chemotherapy for prior oncologic conditions. At DXA evaluation, 45.01% of women were diagnosed with osteoporosis (*n* = 751), 43.5% with osteopenia (*n* = 726), and 11.5% had normal BMD (*n* = 191) (Table 4).

### 3.2. Association Between Skeletal Risk Indicators and Bone Mineral Density

**Figure 2:**
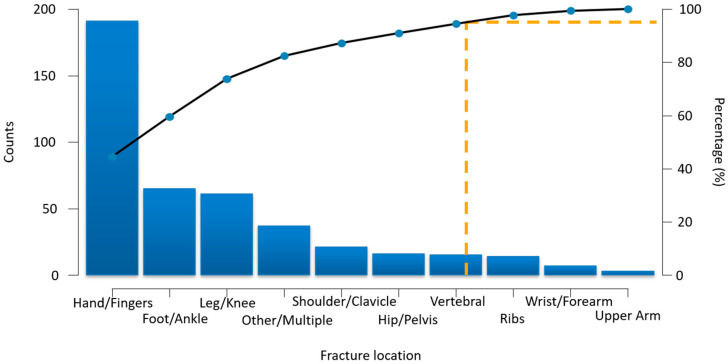
Pareto distribution of self-reported fragility fractures by anatomical site. The dotted yellow line indicates the 95% cumulative frequency threshold, highlighting dominant fracture sites.

Of the total study population, 25.8% of participants (430 out of 1669) reported experiencing at least one personal fragility fracture after the age of 50. The most frequently affected site was the hand/fingers (11.4%), followed by the foot/ankle (3.9%) and knee/leg region (3.7%), which are common locations for low-energy trauma in postmenopausal women. Although less prevalent, hip/pelvis fractures were reported in 0.96% of cases, a proportion of specific clinical relevance due to their impact on functional independence. Other classical osteoporotic sites, such as the vertebrae (0.9%), ribs (0.84%), and shoulder/clavicle (1.26%), were also observed. Wrist/forearm fractures (0.42%) and proximal humerus (0.18%) were the least frequent, possibly reflecting underreporting or variations in fall mechanisms. A total of 2.2% reported multiple or unspecified fractures, indicating a subgroup at higher cumulative risk (Figure 2).

At the time of the assessment, 74.5% of participants showed no radiological evidence of bone lesions. Among those with detectable lesions, the upper extremities were the most frequently affected area, noted in 14.9% of women, followed by the lower extremities (6.3%) and multiple-site lesions (1.9%). Vertebral lesions, although rare (0.8%), may be clinically significant due to their often-asymptomatic nature. The other category (1.6%) likely includes non-specific or less commonly classified lesions.

Regarding family history, only 3.8% of the women (63 out of 1669) reported having first-degree relatives who experienced osteoporotic fractures. The most frequently mentioned event was maternal hip fracture, cited by 45 of the 63 respondents with a positive family history. Others reported vertebral or wrist fractures in mothers or sisters (Table 5).

**Figure 3:**
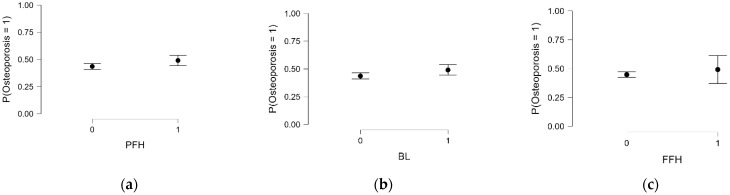
Predicted probability of osteoporosis diagnosis by skeletal fragility indicators: (**a**) personal history of fragility fractures (PFH), (**b**) radiologically observed bone lesions (BL), and (**c**) family history of osteoporotic fractures (FFH). On the x-axis, 0 indicates the absence and 1 the presence of each respective risk factor.

Table 6 presents the association between skeletal fragility indicators, personal history of fragility fractures, radiologically observed bone lesions, and family history of osteoporotic fractures, and osteoporosis diagnosis based on DXA. Women who reported a previous fragility fracture had a noticeably higher prevalence of osteoporosis (49.1%) compared to those without such a history (43.6%). While the difference was modest, it approached statistical significance (χ^2^ = 5.98, *p* = 0.050). This association was further supported by a univariate logistic regression model, which showed that women with a history of fracture had 25% higher odds of being diagnosed with osteoporosis (OR = 1.25; 95% CI: 1.00–1.55; *p* = 0.049), as illustrated in Figure 3a.

A similar trend was observed among women with bone lesions identified on imaging. These participants also showed a higher proportion of osteoporosis diagnoses (49.1% vs. 43.6%) and fewer instances of normal bone mineral density (8.9% vs. 12.4%) when compared to those without lesions. Although the association did not reach statistical significance (χ^2^ = 5.57, *p* = 0.062), logistic regression indicated a borderline effect, with a slight increase in the odds of osteoporosis (OR = 1.24; 95% CI: 0.99–1.56; *p* = 0.051), as shown in Figure 3b.

In contrast, the presence of a family history of osteoporotic fractures did not appear to meaningfully influence the distribution of BMD categories. Women with and without such history showed similar proportions of osteoporosis (49.2% vs. 44.9%), osteopenia (42.9% vs. 43.5%), and normal BMD (7.9% vs. 11.6%). Statistical testing confirmed the absence of a significant association (χ^2^ = 0.98, *p* = 0.608), and this was further supported by the logistic regression findings (OR = 1.19; 95% CI: 0.72–1.34; *p* = 0.493), as depicted in Figure 3c.

### 3.3. Treatment Categories and Their Association with BMD

**Figure 4:**
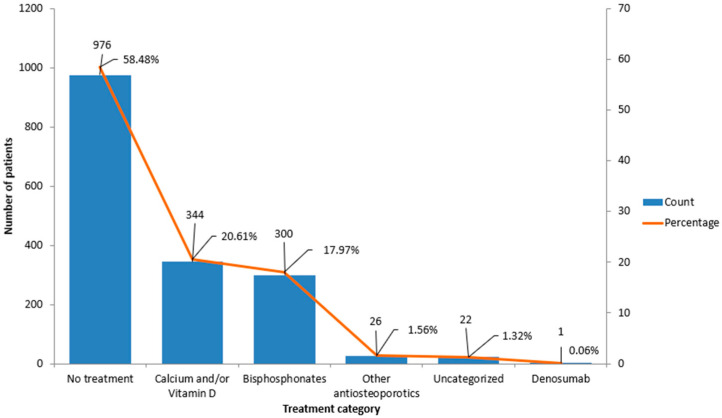
Distribution of study participants by osteoporosis treatment category. Blue bars indicate the number of patients in each treatment group, while the orange line represents the corresponding percentage of the total cohort.

More than half of the women in our study (58.5%, *n* = 976) were not receiving any form of osteoporosis-related treatment at the time of their DXA evaluation. About one in five participants (20.6%, *n* = 344) were taking calcium and/or vitamin D supplements, but without any active medication to protect bone density. Bisphosphonates, the most prescribed antiresorptive drugs, were used by 17.9% (*n* = 300) of the women. A very small number (less than 2%, *n* = 26) were receiving other types of osteoporosis treatments, such as raloxifene, teriparatide, or strontium ranelate, medications that are generally reserved for specific cases or used as alternatives to standard therapies. Denosumab use was extremely rare in our cohort (Figure 4). In total, just over 20% of participants were on any kind of pharmacological therapy specifically aimed at treating osteoporosis, a finding that reflects a significant treatment gap in this vulnerable population.

As shown in Table 7 there were notable differences in bone density status across treatment groups (global χ^2^ test, *p* < 0.001). The highest proportion of osteoporosis was found among women receiving bisphosphonate therapy, with 66.3% meeting the densitometric criteria for the disease. This was followed by the calcium and vitamin D group, where just over half (51.2%) of the women were osteoporotic. In contrast, among those not receiving any treatment, 35.3% were diagnosed with osteoporosis, 47.8% had osteopenia, and 16.8% had normal BMD values. The few women receiving other antiosteoporotic therapies, such as raloxifene or teriparatide, had a high proportion of osteoporosis (73.1%), reflecting the fact that these treatments are often reserved for more severe cases. The single participant treated with denosumab was also osteoporotic. Pairwise comparisons confirmed that osteoporosis was significantly more frequent in the bisphosphonate group compared to both the untreated group (66.3% vs. 35.3%, *p* < 0.001) and the calcium/vitamin D group (66.3% vs. 51.2%, *p* = 0.006). Among all women with confirmed osteoporosis (*n* = 751), the majority (421 women, or 56%) were not receiving any pharmacological treatment at the time of DXA evaluation, highlighting a substantial care gap in this high-risk population.

To better understand real-world treatment patterns, we constructed a multivariate logistic regression model assessing independent predictors of active pharmacologic therapy. The results indicated that bone mineral density (BMD) status was the strongest independent driver of treatment decisions (Table 8). Compared to women with normal BMD, those with osteoporosis were significantly more likely to be receiving active pharmacological therapy (OR = 0.160; 95% CI: [0.080–0.320]; *p* < 0.001), while those with osteopenia also had increased odds of treatment (OR = 0.417; 95% CI: [0.209–0.829]; *p* = 0.013). Age at menopause was also a significant factor. Each additional year in age at menopause was associated with a slightly higher likelihood of not receiving treatment (OR = 1.027; 95% CI: [1.004–1.050]; *p* = 0.022). This suggests that women with earlier menopause might be more closely monitored or considered at higher risk, prompting earlier intervention.

On the other hand, other skeletal fragility indicators, including personal or family history of fractures and radiologic bone lesions, did not show significant associations with treatment initiation in the adjusted model. Similarly, prior hormone replacement therapy, corticosteroid exposure, and BMI did not independently influence treatment status. These findings highlight a potential disconnect between clinical guidelines that emphasize multifactorial risk and real-world prescribing practices, which still tend to prioritize BMD over other important fracture predictors.

**Figure 5:**
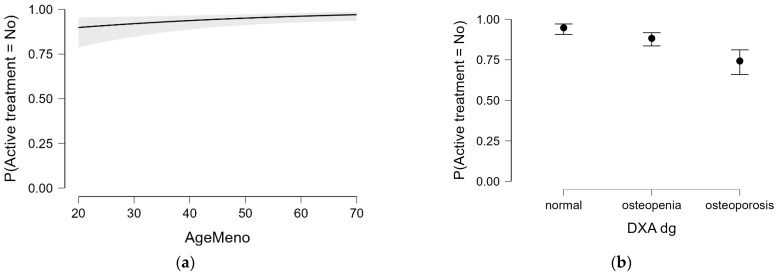
Predicted probability of not receiving active osteoporosis treatment based on independent predictors from the logistic regression model. (**a**) Association with age at menopause (years); (**b**) association with DXA diagnostic category. Shaded areas and error bars indicate 95% confidence intervals.

Figure 5 illustrates the modeled probability of receiving no active osteoporosis treatment based on independent predictors from the logistic regression model. As shown in Figure 5a, women with earlier menopause had a higher probability of being untreated, which may reflect a gap in recognizing the long-term skeletal consequences of early estrogen deprivation. Meanwhile, Figure 5b highlights that DXA-based osteoporosis diagnosis remained the strongest determinant of pharmacologic treatment decisions, with patients meeting densitometric criteria for osteoporosis being significantly less likely to remain untreated. Together, these patterns suggest that treatment decisions are primarily driven by bone density findings, whereas other clinically relevant factors, such as early menopause, may be under-recognized in practice.

### 3.4. Impact of Hormone Replacement Therapy

Although HRT was not associated with bone mineral density status at the time of DXA evaluation (χ^2^ = 0.716, *p* = 0.699), its relationship with skeletal fragility indicators was more nuanced. Women who had received HRT were significantly more likely to report a prior fragility fracture (35.3% vs. 24.4%, *p* < 0.001) and to present radiological signs of skeletal deterioration (34.3% vs. 24.3%, *p* = 0.002) (Table 9).

### 3.5. Independent Predictors of Osteoporosis: A Multivariate Analysis of Clinical and Skeletal Fragility Indicators

To explore independent associations with osteoporosis, we constructed a multivariate logistic regression model incorporating demographic (age, BMI), skeletal (personal and family fracture history, presence of bone lesions), and clinical variables (thyroid dysfunction, diabetes or prediabetes, oncologic history, corticosteroid exposure, and chemotherapy) (Table 10). The model showed a statistically significant fit (χ^2^ = 128.3, *p* < 0.001), with a Nagelkerke R^2^ of 0.099, indicating modest but relevant explanatory capacity.

Age was the strongest predictor: each additional year was associated with a 5.4% increase in the odds of osteoporosis (OR = 1.054; 95% CI: 1.042–1.067; *p* < 0.001). BMI showed an inverse association: each unit increase in BMI reduced the odds by 6.6% (OR = 0.934; 95% CI: 0.914–0.956; *p* < 0.001). Skeletal fragility indicators, personal fracture history (OR = 1.23), family history of fractures (OR = 1.35), and bone lesions (OR = 0.97) did not reach statistical significance (*p* > 0.26), suggesting limited independent contribution in the adjusted model.

Among clinical variables, only diabetes or prediabetes was significantly associated, showing a protective effect (OR = 0.63; 95% CI: 0.45–0.89; *p* = 0.009). Thyroid disease, prior cancer, corticosteroid therapy, and chemotherapy were not significantly related to osteoporosis risk.

**Figure 6:**
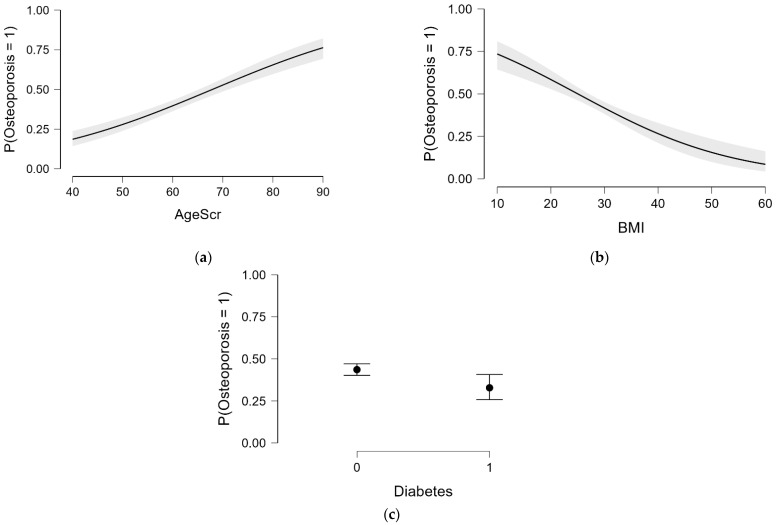
Predicted probability of osteoporosis based on independent predictors from the logistic regression model: (**a**) association with age (years), showing increased risk with advancing age; (**b**) association with body mass index (BMI, kg/m^2^), revealing an inverse correlation; (**c**) association with diabetes status, indicating a lower predicted osteoporosis risk. Shaded areas and error bars indicate 95% confidence intervals.

Figure 6 illustrates the modeled probability of osteoporosis based on independent predictors from the logistic regression model. As shown in Figure 6a, age was positively associated with osteoporosis risk, with a sharp increase across the late menopausal decades. Figure 6b shows an inverse relationship with BMI, reflecting the known protective role of higher body mass on bone mineral density. Interestingly, Figure 6c suggests that women with type 2 diabetes or prediabetes had a lower modeled probability of osteoporosis, which may reflect confounding effects such as increased body weight or altered bone remodeling patterns associated with insulin resistance. These patterns highlight the multifactorial nature of osteoporosis risk, beyond bone density alone.

## 4. Discussion

The present study evaluated skeletal risk indicators and treatment patterns in a large cohort of postmenopausal women referred for DXA screening in a Romanian tertiary care center. By integrating densitometric, clinical, and radiological data, we aimed to identify predictors of osteoporosis diagnosis and treatment, with a particular focus on fragility fractures, bone lesions, and prior hormone therapy. Our findings reveal a substantial gap between clinical risk and therapeutic intervention, highlighting the underutilization of pharmacologic treatment even among high-risk patients.

Our cross-sectional analysis revealed that nearly 90% of the subjects had either osteoporosis or osteopenia, based on DXA measurements. This exceptionally high rate likely reflects a referred population with pre-existing clinical risk factors, but also underscores the extent of low bone density among postmenopausal women undergoing evaluation in Romania. National data indicate that only 15–20% of women over 50 receive DXA screening, and this is often triggered by symptoms or fragility fractures rather than preventive assessment [15]. Despite the relatively young age of many participants, osteoporosis was already prevalent in 45.01% of cases, a rate that far exceeds national estimates for the general population of women over 50 in Romania (20.5%) [15]. This discrepancy reflects the high-risk nature of our referred cohort and underscores the likelihood that osteoporosis is both underdiagnosed and undertreated in the broader population, particularly in the absence of systematic screening programs. Among the women, those who reported at least one fragility fracture after the age of 50 had a higher prevalence of osteoporosis as measured by DXA (49.1% vs. 43.6%), highlighting the clinical relevance of prior fractures even when statistical significance was not reached. Vertebral fractures, which are frequently asymptomatic, are often missed in clinical care; up to two-thirds go undiagnosed [16,17], despite their strong predictive value for future fractures. In our study, radiological bone lesions were also associated with a greater proportion of osteoporosis diagnoses, suggesting that structural changes visible on standard X-rays may signal underlying fragility. Although imaging was performed based on clinical needs such as pain or trauma, many of these radiographs revealed vertebral compressions or cortical thinning, subtle but meaningful markers of skeletal compromise [18,19]. These observations reinforce the importance of integrating radiological findings into the broader assessment of fracture risk, especially in settings where DXA may underestimate fragility. Recent guidelines support this direction, encouraging the use of vertebral fracture assessment (VFA) and other tools to refine diagnosis beyond BMD values alone [20,21].

Only a small proportion of women in our cohort reported a family history of osteoporotic fractures, a factor widely recognized as a genetic contributor to skeletal fragility. Although our analysis did not find a significant association, likely due to underreporting or limited statistical power, this variable remains clinically relevant and is routinely included in fracture risk algorithms such as FRAX [22,23,24]. The absence of a clear link in our study does not diminish its importance in clinical decision-making, but rather highlights the need for more detailed family history collection in routine care.

One of the most striking findings in our study was the high rate of undertreatment. More than half of women with a self-reported fragility fracture were not receiving any pharmacologic therapy, at the time of evaluation. This observation mirrors both European patterns and national data, placing Romania among the countries with the highest treatment gaps in high-risk women [5,25]. Despite clear evidence that fracture risk depends on multiple factors, not just BMD, our data confirm that treatment decisions in practice are still overwhelmingly driven by DXA results. This reliance on T-score thresholds may contribute to the invisibility of patients who do not meet densitometric criteria but are nonetheless at elevated risk.

Another noteworthy finding from our predictive model was the inverse association between type 2 diabetes mellitus and the diagnosis of osteoporosis. Although seemingly paradoxical, this relationship has been consistently reported in the literature. Patients with type 2 diabetes often have normal or even elevated BMD values, likely influenced by higher body weight and the anabolic effects of insulin on bone tissue. Yet, despite this, their actual fracture risk remains elevated, pointing to deeper structural issues not captured by standard DXA assessments [26]. This mismatch underscores an important limitation of relying solely on T-scores when evaluating fracture risk in diabetic patients. Bone quality, cortical porosity, and altered bone turnover in diabetes are not reflected in BMD values, which may lead to false reassurance and missed opportunities for prevention. Our findings support growing calls for tailored risk assessment tools in this population, beyond traditional densitometry.

Other comorbidities known to affect bone metabolism, such as thyroid dysfunction, chronic corticosteroid exposure, and a history of cancer, were also evaluated in our multivariate model. However, none reached statistical significance as independent predictors of osteoporosis diagnosis. This may reflect their relatively low prevalence in our sample or the variability in disease duration and treatment intensity. Nonetheless, these conditions are well-established contributors to skeletal fragility and are consistently recognized in clinical guidelines. For example, long-term glucocorticoid use is a major secondary cause of osteoporosis, known to inhibit osteoblast function and increase bone resorption [27]. Similarly, hyperthyroidism accelerates bone turnover and reduces bone mineral density, even in subclinical forms [28]. Cancer treatments such as chemotherapy and hormone suppression also adversely impact bone health through direct and indirect mechanisms [29]. The lack of observed associations in our cohort should not preclude clinical vigilance, but rather underscores the need for larger, prospective studies to better capture the nuanced effects of these factors on fracture risk and treatment allocation.

Earlier age at menopause, a known contributor to accelerated bone loss, was associated with a greater likelihood of receiving treatment, possibly reflecting heightened clinical awareness of early estrogen deprivation [30]. However, other variables such as prior HRT, corticosteroid use, and family history of fracture had no detectable impact, suggesting that broader fracture risk models (e.g., FRAX-based algorithms) may not yet be consistently applied in practice. Findings related to HRT merit special attention. While HRT is known to prevent early postmenopausal bone loss, its protective effects are not sustained after discontinuation. In our cohort, prior use of HRT was not associated with improved bone mineral density at the time of DXA evaluation and was paradoxically linked to a higher prevalence of fragility fractures. This may reflect residual confounding. Women with greater baseline fracture risk may have been more likely to receive HRT for climacteric symptoms, without structured bone follow-up. These findings support recommendations to transition former HRT users to targeted osteoporosis therapy if risk remains elevated [31,32,33]. In the context of our study’s broader treatment gap, the HRT–fracture relationship highlights a missed opportunity for long-term bone protection.

Romania faces one of the largest osteoporosis treatment gaps in Europe, with a recent national estimate indicating that up to 78% of high-risk women remain untreated, one of the highest rates reported among EU countries [15,34]. Although we did not assess the reasons directly, the previous literature cites limited awareness, fear of adverse effects, and access-related barriers as key contributors [33,35,36]. This disconnect between risk and treatment uptake highlights an urgent need for coordinated post-fracture care pathways and targeted public health strategies focused on prevention. Educational efforts targeting both healthcare providers and patients could play a critical role in reducing avoidable fractures [37,38,39]. Moreover, studies consistently show that early pharmacologic intervention is not only clinically effective but also cost-saving in the long term, by reducing hospitalizations and disability associated with osteoporotic fractures [40,41].

Evidence from large trials supports the efficacy of antiresorptive and anabolic therapies in reducing vertebral and non-vertebral fractures [42,43,44]. In our study, pharmacologic treatment was strongly associated with lower BMD values, suggesting that clinicians continue to prioritize densitometric thresholds when initiating therapy. Conversely, prior use of HRT did not appear to confer lasting protection, highlighting that estrogen’s skeletal benefits are short-lived in the absence of follow-up antiresorptive therapy. Moreover, current guidelines do not recommend HRT solely for osteoporosis prevention, due to associated cardiovascular and oncological risks [33]. In contrast, bisphosphonates and denosumab are typically prescribed to women with already established, often severe osteoporosis. For example, bisphosphonates (such as alendronate, risedronate, and zoledronate) reduce the risk of vertebral fractures by 60–70%, hip fractures by 40–50%, and non-vertebral fractures by approximately 20–30% [45]. Denosumab, when used longterm, has been shown to significantly increase BMD and maintain low rates of both vertebral and non-vertebral fractures [46,47]. These therapies remain the cornerstone of fracture prevention in women with established osteoporosis or prior fragility fractures, when no contraindications exist, and their timely initiation is critical in reducing the cumulative burden of osteoporotic disease.

Additionally, we examined whether residential background influenced therapeutic decisions. Urban or rural residence was not significantly associated with active treatment uptake, indicating that in this referred cohort, geographic location did not independently impact management decisions. However, this finding should be interpreted with caution. We acknowledge that structural disparities in healthcare access, particularly in rural areas, likely remain a broader issue in Romania [48]. These may include delayed referral, fewer DXA facilities, and lower patient awareness, elements that fall outside the scope of our current dataset but warrant further investigation in population-based studies. These challenges are further reflected in the broader European benchmarking data. According to the 2021 SCOPE report, Romania ranked last among 29 countries in terms of osteoporosis-related disease burden, while scoring mid-range (14th) in healthcare provision metrics, including policy framework and service uptake [15]. This apparent mismatch between service availability and clinical outcomes underscores potential barriers in translating healthcare capacity into effective prevention and treatment at scale.

Several limitations of this study should be acknowledged to contextualize the findings. Firstly, the cross-sectional design does not allow for causal inference. For example, the observed association between treatment status and osteoporosis reflects clinical practice; treatment typically follows diagnosis, not the reverse. Secondly, fracture history was based on self-report, which may lead to underreporting, especially of minor or asymptomatic vertebral fractures. The small number of patients with a positive family history also limits our ability to detect statistically significant associations for this variable; future studies could strengthen this aspect by expanding the sample size. Additionally, we lacked longitudinal data on treatment duration, BMD evolution under therapy, and the timing of fractures relative to treatment initiation. This limits our ability to directly assess treatment effectiveness over time. Moreover, due to the retrospective nature of the data collection, we were unable to determine the precise timing of certain clinical events, such as the onset or duration of hormone replacement therapy, or the exact moment of fracture or bone lesion occurrence. Similarly, imaging studies (e.g., X-rays indicating bone lesions) were not performed systematically, but rather as part of routine care. As such, variability in the clinical indication or radiological technique may have introduced heterogeneity into the assessment of skeletal fragility. These temporal and procedural inconsistencies limit our ability to fully standardize exposure–outcome relationships across participants. Furthermore, our cohort includes a relatively large proportion of younger postmenopausal women (ages 40–59), which may not fully reflect the age distribution of the general postmenopausal population. However, this composition reflects real-world clinical referrals, where DXA is often performed based on individual risk profiles rather than age alone. While this may limit external generalizability, it enhances the clinical relevance of our findings in early screening and intervention contexts.

Despite these limitations, the study’s strengths include a relatively large and representative cohort and the use of real-world clinical data, offering a realistic picture of local clinical practice and challenges. To our knowledge, this is one of the most extensive Romanian analyses exploring both skeletal risk indicators and treatment patterns in this vulnerable population. By integrating DXA data with clinical factors, fracture history, radiographic findings, and hormone therapy records, the study provides a multidimensional view of osteoporosis care, one that reflects both diagnostic realities and treatment gaps in practice. The use of multivariate models further strengthened our ability to isolate independent predictors of diagnosis and therapy, pointing to actionable targets for improving care delivery. Importantly, our findings emphasize that many women at high risk remain untreated despite evident fragility signs, and that therapeutic decisions often rely disproportionately on BMD thresholds. Our findings highlight the critical need to strengthen risk-based approaches to osteoporosis management, particularly in Romania, where underdiagnosis and undertreatment remain significant challenges in routine clinical practice.

## 5. Conclusions

Our investigation reinforces the understanding that postmenopausal osteoporosis is a complex, multifactorial condition. While bone mineral density remains a cornerstone of diagnosis, it does not fully capture an individual’s fracture risk. Clinical factors such as a personal history of fragility fractures, family history of osteoporosis, and imaging-detected bone lesions all contribute to an individual’s skeletal vulnerability, even if not all emerged as independent predictors in our adjusted models. Furthermore, advanced age and lower body weight continue to stand out as robust predictors of severe osteoporosis, underscoring the importance of integrating demographic and clinical information into screening practices. At the same time, our study highlights a persistent and troubling gap in care, as many high-risk women remain untreated despite clearly meeting the criteria for pharmacological intervention. This underlines the need for more proactive identification and timely management of individuals at elevated fracture risk.

## Figures and Tables

**Table 1:** Clarification of categorical variables for skeletal fragility indicators and treatment indicators.

Category	Subcategory/Type	Definition/Description
Personal fractures (PHF)	Foot/ankle	Fractures of the toes, metatarsals, tarsals, or ankle bones
	Hand/fingers	Fractures involving phalanges or metacarpals
	Hip/pelvis	Fractures of the femoral neck, intertrochanteric area, or pelvic ring
	Leg/knee	Fractures of the tibia, fibula, or patella
	Shoulder/clavicle	Clavicular/proximal humeral fractures (excluding upper arm shaft)
	Upper arm	Humeral shaft fractures distinct from shoulder region
	Wrist/forearm	Fractures of the distal radius, ulna, or forearm region
	Vertebral	Clinical or radiographic vertebral body fractures
	Ribs	Fractures of one or more ribs
	Other/multiple	Multiple/unspecified locations; often reported as various/several fractures
	No fracture	No reported personal fragility fracture history
Family history of fracture (FHF)	Hip	First-degree relatives (mother, sister) with a hip fracture, mostly maternal
	Vertebral	Vertebral fracture in first-degree relatives
	Wrist	Wrist or distal radius fracture in first-degree relatives
	No family fracture	No reported history of osteoporosis-related fractures in first-degree relatives
Bone lesions	Vertebral	Imaging-detected vertebral deformity (compression, wedge, etc.)
	Upper extremity	Lesions in humerus, radius, or clavicle observed on imaging
	Lower extremity	Femur or tibia/fibula lesions noted radiographically
	Multiple	Bone lesions present in more than one anatomical region
	Other	Non-specific or poorly localized lesions (e.g., suspected lesion, not otherwise classified)
	No lesion	No bone lesion noted in radiologic or clinical documentation

**Table 2:** Classification of antiosteoporotic treatments by category and representative agents.

Treatment Category	Representative Agents/Examples	Notes
No treatment	—	No supplements or osteoporosis medications reported
Calcium and/or vitamin D	Calcium and/or vitamin D_3_ supplements, including combinations such as calcium carbonate, vitamin D_3_, and fixed-dose calcium + D_3_ complexes	Used as supplements only, without pharmacological antiresorptive agents
Bisphosphonates	Alendronate, risedronate, ibandronate	First-line antiresorptive drugs; oral or injectable; weekly or monthly
RANKL inhibitors	Denosumab	Injectable antiresorptive therapy, alternative to bisphosphonates
Other antiosteoporotics	Raloxifene, teriparatide, strontium ranelate	Second-line or specific case therapies; often used in severe osteoporosis
Uncategorized/unknown	Not clearly specified	Cases where medication data were incomplete or unclear

**Table 3:** Distribution of study participants by age group.

Age Group (Years)	Count	Percentage (%)
40–49	83	4.98
50–59	513	30.77
60–69	674	40.43
70–79	335	20.07
80+	64	3.84

**Table 4:** Baseline demographic and clinical characteristics.

Category	Count	Percentage (%)
Urban residence	1151	68.96
Rural residence	517	30.97
BMI ≥ 25 (overweight/obese)	1268	75.97
BMI 18.5–24.9 (normal)	400	23.97
Thyroid disorders	133	7.97
Type 2 diabetes or prediabetes	250	14.98
History of cancer	66	3.95
Chronic corticosteroid therapy	83	4.97
Chemotherapy	50	3.00
DXA diagnosis		
Osteoporosis	751	45.01
Osteopenia	726	43.50
Normal BMD	191	11.50

BMI, body mass index; DXA, dual-energy X-ray absorptiometry; BMD, bone mineral density.

**Table 5:** Distribution of personal and familial fractures and radiologic lesions.

Category Type	Subtype	Count	Percentage (%)
Personal fracture	No fracture	1239	74.24
	Foot/ankle	65	3.90
	Hand/fingers	191	11.44
	Hip/pelvis	16	0.96
	Leg/knee	61	3.66
	Other/multiple	37	2.22
	Ribs	14	0.84
	Shoulder/clavicle	21	1.26
	Upper arm	3	0.18
	Vertebral	15	0.90
	Wrist/forearm	7	0.42
Family fracture	No family fracture	1606	96.2
	Hip	45	2.7
	Vertebral	12	0.7
	Wrist	6	0.4
Bone lesion	Lower extremity	105	6.3
	Multiple	31	1.9
	No lesion	1243	74.5
	Other	27	1.6
	Upper extremity	249	14.9
	Vertebral	14	0.8

**Table 6:** Distribution of BMD categories by skeletal fragility indicators factors.

Risk Factor		Normal BMD *n* (%)	Osteopenia *n* (%)	Osteoporosis *n* (%)	Total (*n*)	Chi^2^ (*p* Value)
Personal fracture history	No	154 (12.4)	545 (44.0)	540 (43.6)	1239	0.050
	Yes	38 (8.8)	181 (42.1)	211 (49.1)	430	
Bone lesions	No	154 (12.4)	547 (44.0)	542 (43.6)	1243	0.058
	Yes	38 (8.9)	179 (42.0)	209 (49.1)	426	
Family fracture history	No	187 (11.7)	698 (43.5)	719 (44.9)	1604	0.608
	Yes	5 (7.9)	27 (42.9)	31 (49.2)	63	

BMD, bone mineral density. *p*-values reflect comparisons across BMD categories.

**Table 7:** DXA diagnosis by treatment category.

Treatment Category	Normal *n* (%)	Osteopenia *n* (%)	Osteoporosis *n* (%)	Total (*n*)	Chi^2^ (*p* Value)
Bisphosphonates	10 (3.3)	91 (30.3)	199 (66.3)	300	<0.001
Calcium and/or vitamin D	17 (4.9)	151 (43.9)	176 (51.2)	344	0.006
Denosumab	0 (0.0)	0 (0.0)	1 (100.0)	1	-
No treatment	164 (16.8)	467 (47.8)	345 (35.3)	976	<0.001
Other antiosteoporotics	1 (3.8)	6 (23.1)	19 (73.1)	26	0.113
Uncategorized/unknown	0 (0.0)	11 (50.0)	11 (50.0)	22	0.261

**Table 8:** Multivariate logistic regression analysis of predictors associated with active osteoporosis treatment use.

Predictor Variable	Coefficient	OR	95% CI	*p* Value
Intercept	1.559	4.754	1.103–20.491	0.036
Age at menopause (years)	0.026	1.027	1.004–1.050	0.022
BMI (kg/m^2^)	0.003	1.003	0.977–1.030	0.801
Urban residence	−0.016	0.985	0.670–1.448	0.937
Corticosteroid therapy	−0.514	0.598	0.234–1.526	0.282
Hormone replacement therapy	−0.212	0.809	0.561–1.169	0.259
Personal fracture history	−0.172	0.842	0.129–5.505	0.857
Family fracture history	−0.27	0.763	0.415–1.403	0.385
Radiological bone lesion	−0.141	0.869	0.132–5.696	0.883
DXA: osteopenia vs. normal	−0.876	0.417	0.209–0.829	0.013
DXA: osteoporosis vs. normal	−1.83	0.16	0.080–0.320	<0.001

OR, odds ratio; CI, confidence interval; BMI, body mass index; HRT, hormone replacement therapy; BMD, bone mineral density.

**Table 9:** Association between HRT use and bone health parameters.

Outcome		HRT: No *n* (%)	HRT: Yes *n* (%)	Chi^2^ (*p* Value)
DXA diagnosis	Normal	167 (11.4)	25 (12.4)	0.699
	Osteopenia	644 (43.9)	82 (40.8)	
	Osteoporosis	657 (44.8)	94 (46.8)	
Personal fracture history	No	1109 (75.5)	130 (64.7)	<0.001
	Yes	359 (24.5)	71 (35.3)	
Bone lesions	No	1111 (75.7)	132 (65.7)	0.002
	Yes	357 (24.3)	69 (34.3)	
Active treatment	No	1188 (80.9)	154 (76.6)	0.149
	Yes	280 (19.1)	47 (23.4)	

HRT, hormone replacement therapy; DXA, dual-energy X-ray absorptiometry.

**Table 10:** Logistic regression models for predicting osteoporosis.

Predictor Variable	Coefficient	OR	95% CI (OR)	*p* Value
Age (years)	0.053	1.054	1.042–1.067	<0.001
BMI	−0.068	0.934	0.914–0.956	<0.001
Personal fracture history	0.207	1.231	0.244–6.204	0.802
Family fracture history	0.302	1.352	0.794–2.303	0.267
Bone lesions	−0.034	0.967	0.191–4.898	0.968
Thyroid disorders	0.161	1.175	0.933–1.479	0.170
Type 2 diabetes	−0.460	0.631	0.447–0.891	0.009
Cancer history	0.167	1.182	0.725–1.924	0.503
Corticosteroid therapy	0.044	1.045	0.449–2.428	0.919
Chemotherapy exposure	−0.575	0.563	0.282–1.122	0.103

OR, odds ratio; CI, confidence interval; BMI, body mass index.

## Data Availability

The original contributions presented in this study are included in the article. Further inquiries can be directed to the first author.

## Abbreviations

The following abbreviations are used in this manuscript:

	Full term
BMD	Bone mineral density
DXA	Dual-energy X-ray absorptiometry
HRT	Hormone replacement therapy
BMI	Body mass index
OR	Odds ratio
CI	Confidence interval
MHT	Menopausal hormone therapy
PFH	Personal fracture history
FFH	Family fracture history
BL	Bone lesion
SD	Standard deviation
T-score	Standard deviation from young adult reference
FRAX	Fracture risk assessment tool
RANKL	Receptor activator of nuclear factor kappa-B ligand
